# Nucleic Acid Conjugates: Unlocking Therapeutic Potential

**DOI:** 10.1021/acsbiomedchemau.4c00092

**Published:** 2024-12-18

**Authors:** Disha Kashyap, Michael J. Booth

**Affiliations:** † Department of Chemistry, University of Oxford, Mansfield Road, Oxford OX1 3TA, U.K.; ‡ Department of Chemistry, University College London, 20 Gordon Street, London WC1H 0AJ, U.K.

**Keywords:** Nucleic acid therapeutics, antisense oligonucleotides, small interfering RNA, aptamers, splice switching, gene knockdown, bioconjugation, cell delivery, cell targeting, targeted activation

## Abstract

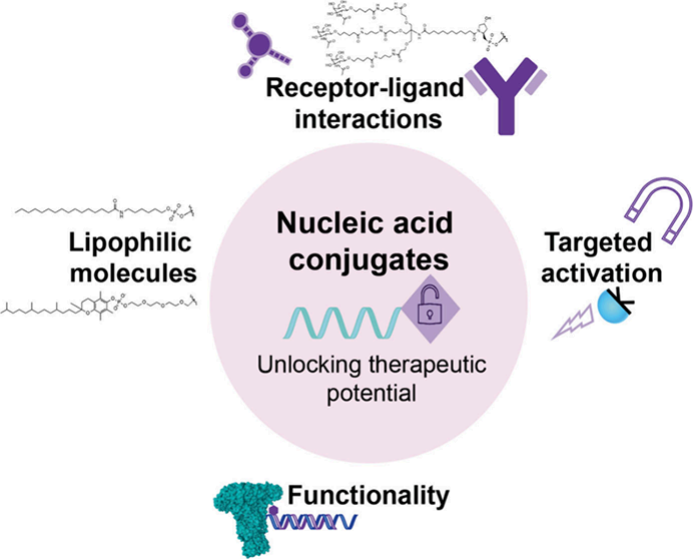

Nucleic acids have emerged as a powerful class of therapeutics. Through simple base pair complementarity, nucleic acids allow the targeting of a variety of pathologically relevant proteins and RNA molecules. However, despite the preliminary successes of nucleic acids as drugs in the clinic, limited biodistribution, inadequate delivery mechanisms, and target engagement remain key challenges in the field. A key area of research has been the chemical optimization of nucleic acid backbones to significantly enhance their “drug-like” properties. Alternatively, this review focuses on the next generation of nucleic acid chemical modifications: covalent biochemical conjugates. These conjugates are being applied to improve the delivery, functionality, and targeting. Exploiting research on heterobifunctionals, such as PROTACs, RIBOTACs, molecular glues, etc., has the potential to dramatically expand nucleic acid drug functionality and target engagement capabilities. Such next-generation chemistry-based enhancements have the potential to unlock nucleic acids as effective and versatile therapeutic agents.

## Introduction

**Figure 1:**
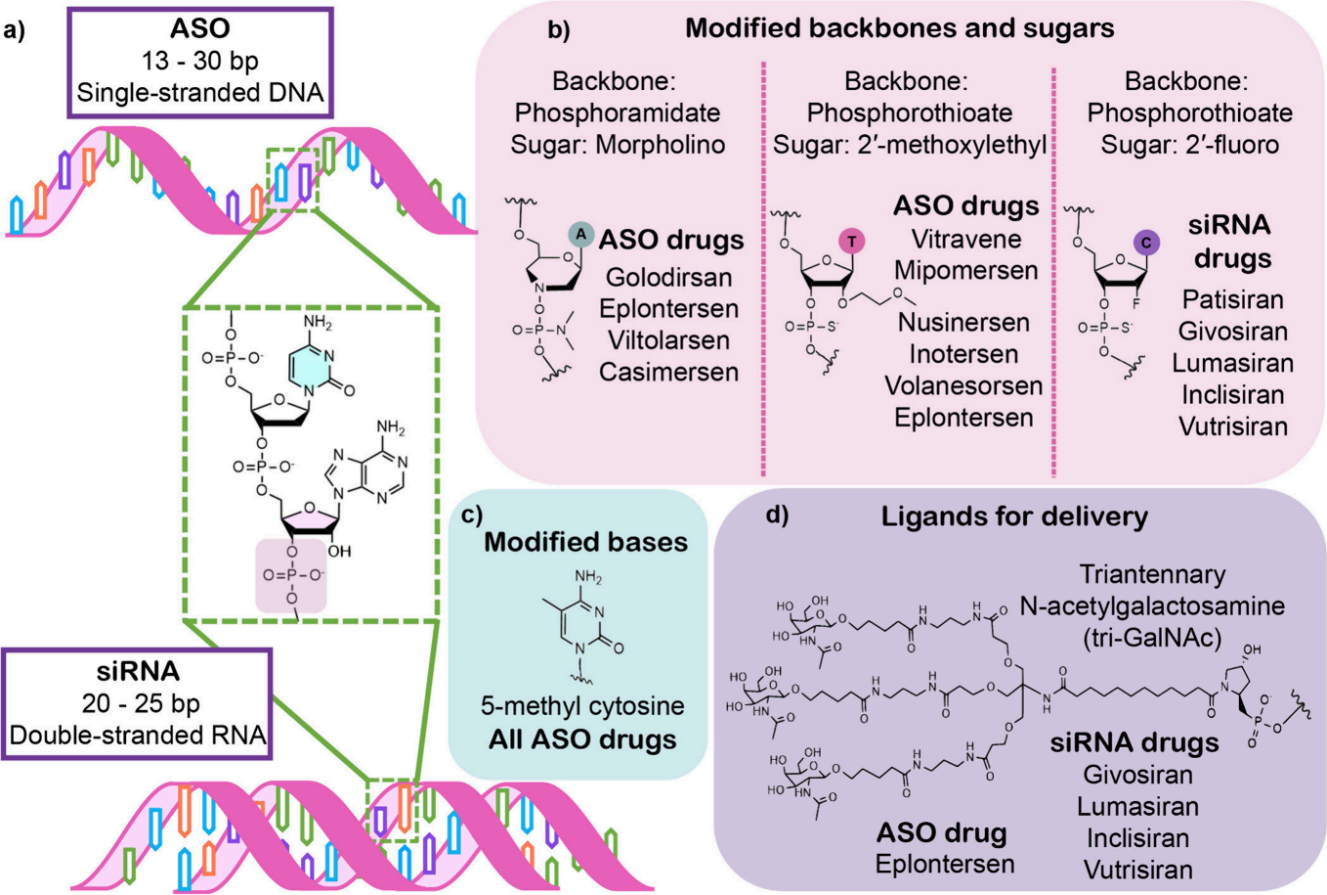
Approved ASO/siRNA nucleic acid therapeutics on the market and their optimized chemistries. a) Antisense oligonucleotides (ASOs) are single stranded and small interfering RNA (siRNA) are double stranded. Strucute chemical modifications in the b) backbone/sugar and c) cytosine base present in the listed approved ASO/siRNA nucleic acid therapeutics. d) Enhanced hepatic delivery achieved via the tri-GalNAc moiety through receptor–ligand interactions in listed approved ASO and siRNA drugs.

Nucleic acid therapeutics are a class of drugs comprising primarily DNA or RNA as the therapeutically active agent. To date, a total of 24 nucleic acid drugs have been approved by the FDA/EMA.^1^ While the headlines have been taken by the mRNA (mRNA) and CRISPR-Cas-based therapeutics, the majority of nucleic acid therapeutics are based on short oligonucleotides that can be administered without additional delivery agents. This review will focus on these shorter species, which include short-interfering RNA (siRNAs), antisense oligonucleotides (ASOs), aptamers, and CpG deoxynucleotides. They are already used in the clinic for treating a variety of diseases, including Duchenne’s Muscular Dystrophy, Spinal Muscular Atrophy, Transthyretin amyloidosis, and Hypercholesterolemia (Table 1).^2^ Here, we give a condensed description of their therapeutic applications and the state-of-the-art in their chemical modification (Figure 1), which have been extensively reviewed by others.^3,4^ This is followed by a main focus on the exciting development of nucleic acid conjugates, i.e., nucleic acids covalently linked to other molecules, which have been shown to give nucleic acids a vast array of new abilities. While nucleic acid delivery is commonly carried out with the formation of noncovalent, electrostatically formed nanoparticles, especially *in vitro*,^5,6^ it is covalent conjugates that are being established as the next generation of nucleic acid therapeutics, with the potential to move beyond just nucleic acid delivery.

**Table 1:** List of Approved ASO, siRNA, Aptamer, and CpG Nucleic Acid Therapeutics^a^

ID	Drug Name/Year	Target/Indication	Chemistry	Mechanism of action
1	VITRAVENE^21^ (fomivirsen) 1998	CMV IE-2, CMV retinitis	21-mer ASO Backbone: full PS	RNase H
2	MACUGEN^49^ (pegaptanib) 2004	VEGF165, neovascular AMD	27-mer RNA aptamer Sugars: 2′-OH, 2′-F, 2′-OMe Backbone: full PO Conjugate: 5′-PEG	Protein binding
3	KYNAMRO^43^ (mipomersen) 2013	ApoB-100, homozygous familial hypercholesterolemia (HoFH)	20-mer ASO Backbone: full PS Sugars: 2′-O-MOE, 2′-H (gapmer)	RNase H
4	EXONDYS 51^26^ (eteplirsen) 2016	Dystrophin, Duchenne muscular dystrophy (DMD)	30-mer ASO Backbone: full PMO	Exon exclusion
5	SPINRAZA^41^ (nusinersen) 2016	SMN2, spinal muscular atrophy (SMA)	18-mer ASO Backbone: full PS Sugars: 2′-MOE	Exon inclusion
6	ONPATTRO^50^ (patisiran) 2018	TTR, hereditary Transthyretin Amyloidosis (hATTR)	21-mer siRNA Backbone: full PO Sugars: 2′-OH, 2′-OMe, 2′-H	RISC/Ago2
7	TEGSEDI^44^ (inotersen) 2018	TTR, hATTR	20-mer ASO Backbone: full PS Sugars: 2′-MOE/2′-H (gapmer)	RNase H
8	GIVLAARI^48^ (givosiran) 2019	ALAS1, acute hepatic porphyria	21-mer/23-mer siRNA Backbone: PO, PS Sugars: 2′-OMe, 2′-F Conjugate: 3′-tri-GalNAc	RISC/Ago2
9	VYONDYS 53^27^ (golodirsen) 2019	Dystrophin, DMD	25-mer ASO Backbone: full PMO	Exon exclusion
10	WAYLIVRA^45^ (volanesorsen) 2019	Apolipoprotein CIII (apoCIII), familial chylomicronemia syndrome (FCS)	20-mer ASO Backbone: full PS Sugars: 2′-MOE/2′-H (gapmer)	RNase H
11	OXLUMO^51^ (lumasiran) 2020	Hydroxyacid oxidase 1 (HAO1), primary hyperoxaluria type 1 (PH1)	21-mer/23-mer siRNA Backbone: PO, PS Sugars: 2′-OMe, 2′-F Conjugate: 3′-tri-GalNAc	RISC/Ago2
12	LEQVIO (inclisiran)^52^ 2020 (EMA) 2021 (FDA)	PCSK9, heterozygous familial hypercholesterolemia (HeFH) or clinical atherosclerotic cardiovascular disease (ASCVD)	21-mer/23-mer siRNA Backbone: PO, PS Sugars: 2′-OMe, 2′-F, 2′-H Conjugate: 3′-tri-GalNAc	RISC/Ago2
13	VILTEPSO^28^ (viltolarsen) 2020	Dystrophin, DMD	21-mer ASO Backbone: full PMO	Exon exclusion
14	AMONDYS 45^29^ (casimersen) 2021	Dystrophin, DMD	22-mer ASOBackbone: full PMO	Exon exclusion
15	CpG 1018, part of HEPLISAV-B^53^ 2017	Adjuvant, Hepatitis B vaccine	22-mer DNABackbone: PS	Immuno-stimulation
16	AMVUTTRA^54^ (vutrisiran) 2022	TTR, hATTR	21-mer/23-mer siRNA Backbone: PO, PS Sugars: 2′-OMe, 2′-F Conjugate: 3′-tri-GalNAc	RISC/Ago2
17	WAINUA^55^ (eplontersen) 2023	TTR, Polyneuropathy Transthyretin Amyloidosis	20-mer ASO Backbone: PO, PS Sugars: 2′-MOE/2′-H (gapmer) Conjugate: 5′-tri-GalNAc	RNase H
18	IZERVAY^56^ (avacincaptad pegol) 2023	Complement protein C5, Geographic autophagy	39-mer RNA aptamer Sugars: 2′-OH, 2′-F, 2′-OMe Backbone: full PO Conjugate: 5′-PEG	Protein binding

a When not stated, the sugar chemistry is full 2′-H, apart from PMOs that have altered sugars themselves. PO, phosphate; PS, phosphorothioate; PMO, phosphorodiamidate morpholino oligomer; OMe, methoxyl; MOE, methoxyethyl; PEG, polyethylene glycol; GalNAc, N-acetylgalactosamine.

### Mechanism of Action and Therapeutic Potential

Short-interfering RNAs (siRNAs) and antisense oligonucleotides (ASOs) share some commonality in their respective mechanisms of action, i.e., the molecular events of the nucleic acid drugs’ activities can be broadly divided into three phases centered around RNA binding: prehybridization, hybridization, and posthybridization.^7^ Prehybridization refers to events leading to the subcellular uptake, localization, and distribution of nucleic acids to achieve effective local concentrations for biological activity. Hybridization is a complex process with much of the cellular machinery not fully characterized;^8^ this holds true especially for ASOs but less so for siRNA. However, factors affecting the potency and specificity of hybridization events such as RNA sequence, secondary structure, and half-life are understood well for both. Posthybridization events result in the pharmacological effect observed and may be enzymatic or noncatalytic.^9^

#### Short Interfering RNAs

Short interfering RNAs (siRNAs) are short, synthetic double-stranded RNA sequences (Figure 1a) that work through a natural enzymatic cascade known as RNA interference (RNAi).^10^ The first step of RNAi is the cleavage of longer double-stranded RNA into siRNAs, containing a two-nucleotide overhang on the 3′ ends of each strand, by the enzyme Dicer. Natural and synthetic siRNA is then taken up into a multiprotein complex, RISC (RNA-induced silencing complex) consisting minimally of Dicer, Tar RNA Binding Protein (TRBP) and Ago2.^11^ Within this complex, the antisense 5′-strand is retained and used for hybridization to target mRNA. Ago2 is the most crucial catalytic component of the RISC, responsible for target mRNA cleavage, the rate-determining step of RNAi.^12^ Therapeutic siRNA can hence be optimized to cleave target specific mRNA, leading to the “knockdown” of the encoded protein.

#### Antisense Oligonucleotides

Antisense oligonucleotides (ASOs) are short, synthetic single-stranded species (Figure 1a) that lead the race as the most clinically viable nucleic acid therapeutics, with nine approved drugs on the market, mainly targeting Duchenne’s Muscular Dystrophy and Spinal Muscular Atrophy, diseases previously thought to be incurable.^1^ Antisense drugs work by altering the metabolism of RNAs. ASOs can degrade or trigger the synthesis of precursor RNAs or mature mRNAs via base complementarity through a variety of mechanisms broadly classed as enzyme-active and splice-switching.^7^

Enzyme-active ASOs (composed of DNA) rely upon RNase-H-dependent mechanisms for targeted mRNA reduction. The RNA-DNA duplex acts as a substrate for RNase H, a response dependent on the oligonucleotide backbone closely resembling DNA.^13^ Ago-2 activating ASOs or “single-stranded siRNA” have also been synthesized with modified chemistries for activating RNAi Ago-2 mediated mRNA cleavage.^14^ Splice-switching or enzyme-inactive ASOs possess backbones invisible to this enzymatic machinery and thus, mask specific oligonucleotide sequences altering splicing decisions, reading frames, and other key steps in translation.^15^ Splice-switching ASOs, thus, can be used to restore the function of a defective gene, inhibit nonsense-mediated decay, and increase transcript stability. The advantage here is that these mechanisms go beyond the canonical mechanism of targeted RNA degradation.

Aptamers and CpG deoxynucleotides have unique mechanisms of action vastly different from that of siRNAs or ASOs.

#### Aptamers

Aptamers are three-dimensional DNA or RNA structures selected from a larger pool of sequences to bind a specific target, a small molecule or protein.^16^ Although similar to antibodies in their high affinities for their target, they possess significant advantages over their protein counterparts such as ease of screening and synthesis, smaller size, and increased permeability and stability.^17^

#### CpG Deoxynucleotides

CpG deoxynucleotides are short single-stranded DNAs that produce an immunostimulatory response. This is known to activate dendritic cells and B cells by interacting with Toll-like receptor 9 resulting in a proinflammatory cascade.^18^ Thus, CpG oligodeoxynucleotides can be used as adjuvants for amplifying immune responses in specific disease contexts.

## Chemical Modification of Nucleic Acids

On the face of it, DNA and RNA do not look like the ideal drug candidates; they are large polyanionic molecules that are unable to diffuse passively across the anionic lipid membranes of cells (unlike small molecules), they are immunostimulatory (antiviral responses), and have poor pharmacokinetic and pharmacodynamic properties. However, these past few decades have seen the development of chemical modifications to increase efficacy, stability, and safety profiles, allowing their transformation into breakthrough medicines (Figure 1, Table 1).

### Backbone Modifications

A key charged analogue of the phosphate backbones, the phosphorothioate linkage, represented the first-generation of nucleic acid therapeutic backbones modifications (Figure 1b).^19^ In phosphorothioate linkages, the introduction of the sulfur atom results in reduced susceptibility to nucleases. Moreover, sulfur has a more diffused charge leading to increased hydrophobicity and increased binding affinity for serum proteins, improving their pharmacological profile. This increased interaction with plasma and intracellular proteins conferred by the PS backbone delays renal clearance. Thus, the first-generation PS ASOs are stable in plasma and tissues with a half-life of 2–3 days.^20^ The first ever FDA/EMA-approved nucleic acid therapeutic, vitravene,^21^ was a fully phosphorothioated ASO for cytomegalovirus retinitis. Furthermore, all RNase H-active ASO therapeutics on the market contain phosphorothioate backbones.

While increased protein interactions of the PS backbone increase circulation time, certain strong protein interactions mediated by the PS backbone can also result in significant toxicity. Moreover, off-target hybridization and subsequent, RNase H-mediate knockdown of essential transcripts is also considered a significant drawback of the PS backbone.^20^ To remove the limitation of the highly charged backbone, uncharged analogues have been developed, including methylphosphonates^22^ and morpholinos^23^ (although, morpholinos also contain altered sugars, morpholino rings replacing the deoxyribose sugars) (Figure 1). These uncharged phosphate ester or phosphoramidate backbones (Figure 1b) show complete nuclease resistance and improved, yet imperfect delivery. Moreover, they operate through an entirely different mechanism of action, the pre-mRNA level by affecting splicing outcomes as these backbones are not recognized by RNase H. This lack of charge, however, results in poor serum-protein binding ability. The reduced protein interactions result in more rapid serum clearance and thus, less favorable pharmacokinetic properties–requiring higher doses for in vivo efficacy.^24^ Another promising, uncharged analogue is the peptide nucleic acids, where the phosphate backbone is replaced by aminoethylglycine units linked by amide bonds (PNAs).^25^ PNAs possess the same merits and pitfalls as other uncharged analogues, although with an even lower aqueous solubility. Morpholino phosphoramidate backbones have been the breakthrough in this area, and are the basis of multiple FDA/EMA-approved nucleic therapeutics for treating Duchenne’s muscular dystrophy (via exon skipping): eteplirsen^26^ (dystrophin, exon 51), golodirsen^27^ (dystrophin, exon 53), viltolarsen^28^ (dystrophin, exon 53), and casimersen^29^ (dystrophin, exon 45).^30^ This is because, compared to other uncharged backbone analogues like the PNAs, PMOs are considered to be more resistant to enzymatic degradation, possess higher aqueous solubility and sequence specificity.^23,31^ Another case of the morpholino sugar is the updated thiomorpholino oligonucleotide (TMO).^32^ TMOs contain morpholino sugars combined with a phosphorothioate backbone. TMOs have been tested for exon skipping in a Duchenne muscular dystrophy (DMD) H2K mdx mouse model and perform well at low concentrations, allowing for safer dosage profiles.

Ultimate conformational preorganization-driven entropy gains in duplex stability are obtained through locked or frozen sugar orientations such as those in locked nucleic acids, LNAs (2′-4′ methylene/ethylene bridges) or tricyclo^33^/bicyclo^34^-(containing sugars fused with cycloalkanes) nucleic acids, tcDNA. While tricycloDNA ASOs are undergoing clinical evaluation for the treatment of Duchenne’s Muscular Dystrophy, several different locked nucleic acid ASOs were shown to be toxic, causing thrombocytopenia, sever liver toxicity, and nephrotoxicity resulting in no ongoing clinical trials for this chemistry.^35^

### Nucleobase and Sugar Modifications

Alterations on the nucleobase and sugar are considered to be key second-generation nucleic acid therapeutic modifications, especially 2′-O-methylated (2′-OMe)^36^ and 2′-fluoro (2′-F)^37^ RNA sugar analogues, and 5-methylcytosine (Figure 1b,c). Development of these modifications was motivated by increasing the potency (2′-OH modifications) and reducing the immunogenic (5-methyl cytosine^38^) properties of the nucleic acid drugs. RNA duplexes are more stable than their DNA counterparts, as the 2′-hydroxyl prefers the axial position, and the steric and stereoelectronic effects lead to the C3′-endo sugar conformation^39^ that results in higher annealing temperatures and greater binding affinity. The conformational equilibrium favors the C3′-endo position over the C2′-endo position, especially in the presence of a highly electronegative 2′-substituent like fluorine.^40^

2′-RNA sugar modifications such as 2′-OMe or 2′-F are not recognized by RNase H but are active through splice modulating mechanisms, as seen in the case of approved drug, nusinersen,^41^ a fully 2′-OMe ASO (exon inclusion) for treating spinal muscular atrophy. In order to obtain increased binding efficacy but retain RNase H activity, the “gapmer” design^42^ was invented and has been applied to many approved nucleic acid drugs. Clinically approved gapmers include mipomersen,^43^ for treating homozygous familial hypercholesterolemia, inotersen,^44^ for treating hereditary transthyretin amyloidosis, and volanesorsen,^45^ for treating familial chylomicronemia syndrome. The “gapmer” design features a central stretch of DNA oligonucleotides (RNase H-active) flanked on either side by 3–5 bp with 2′-modified sugars (RNase H-inactive), improving the nuclease stability and target binding. Approved siRNA drugs such as patisiran^46^ for treating hereditary transthyretin amyloidosis (lipid nanoparticle formulation) also contains 2′-OMe modifications, and both lumasiran,^47^ for treating primary hypoxaluria type 1, and givosiran,^48^ for treating acute hepatic porphyria, contain both 2′-OMe and 2′-F modifications.

## Improving Activity through Conjugation

**Figure 2:**
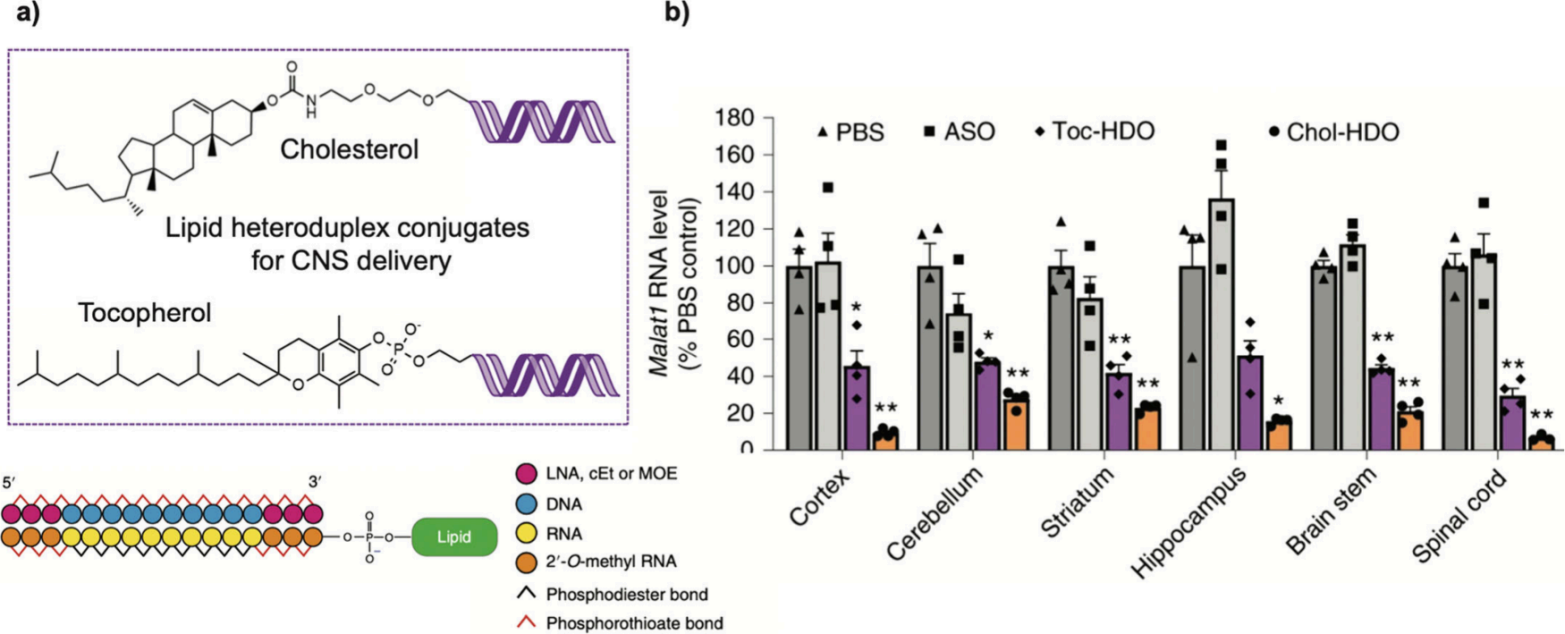
Lipid-modified heteroduplex ASOs (HDOs) for knockdown across the central nervous system (CNS). a) Chemistry of cholesterol- (Chol-) and tocopherol (Toc-) HDOs. b) Widespread knockdown of MALAT1 across the CNS with Chol- and Toc-HDOs. Reprinted (adapted) with permission from ref (75). Copyright 2024 Springer Nature

**Figure 3:**
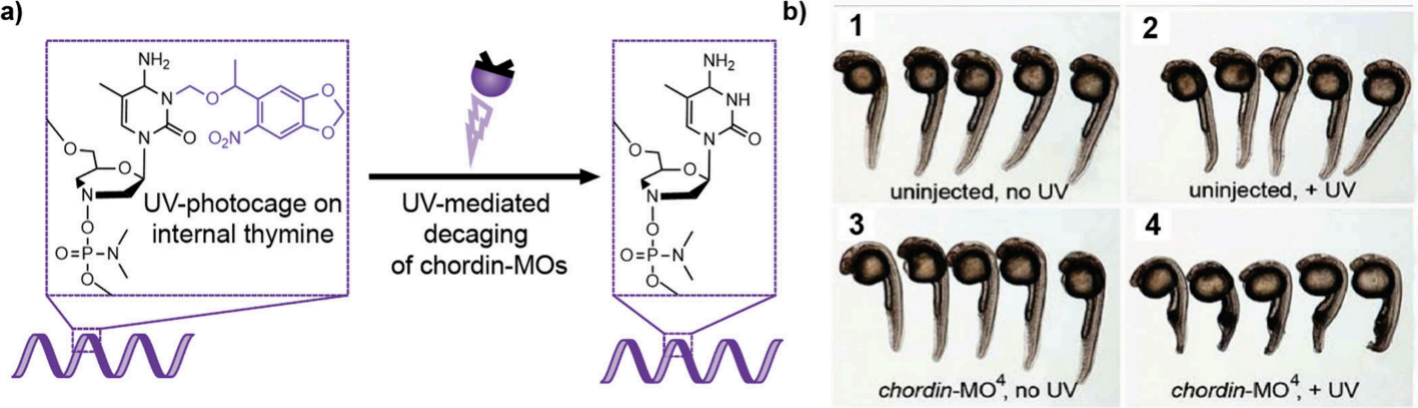
Light-activated control of ASOs in an animal model. a) A nitrobenzyl-photocage was used to inhibit activity of a PMO-based ASO. Activation of the *chordin* PMO, was achieved using ultraviolet (UV) light. b) UV-irradiation resulted in active *chordin* PMO that disrupted endogenous levels of *chordin* resulting in deformity (4), compared to controls (1–3). Reprinted (adapted) with permission from ref (104). Copyright 2024 American Chemical Society.

**Figure 4:**
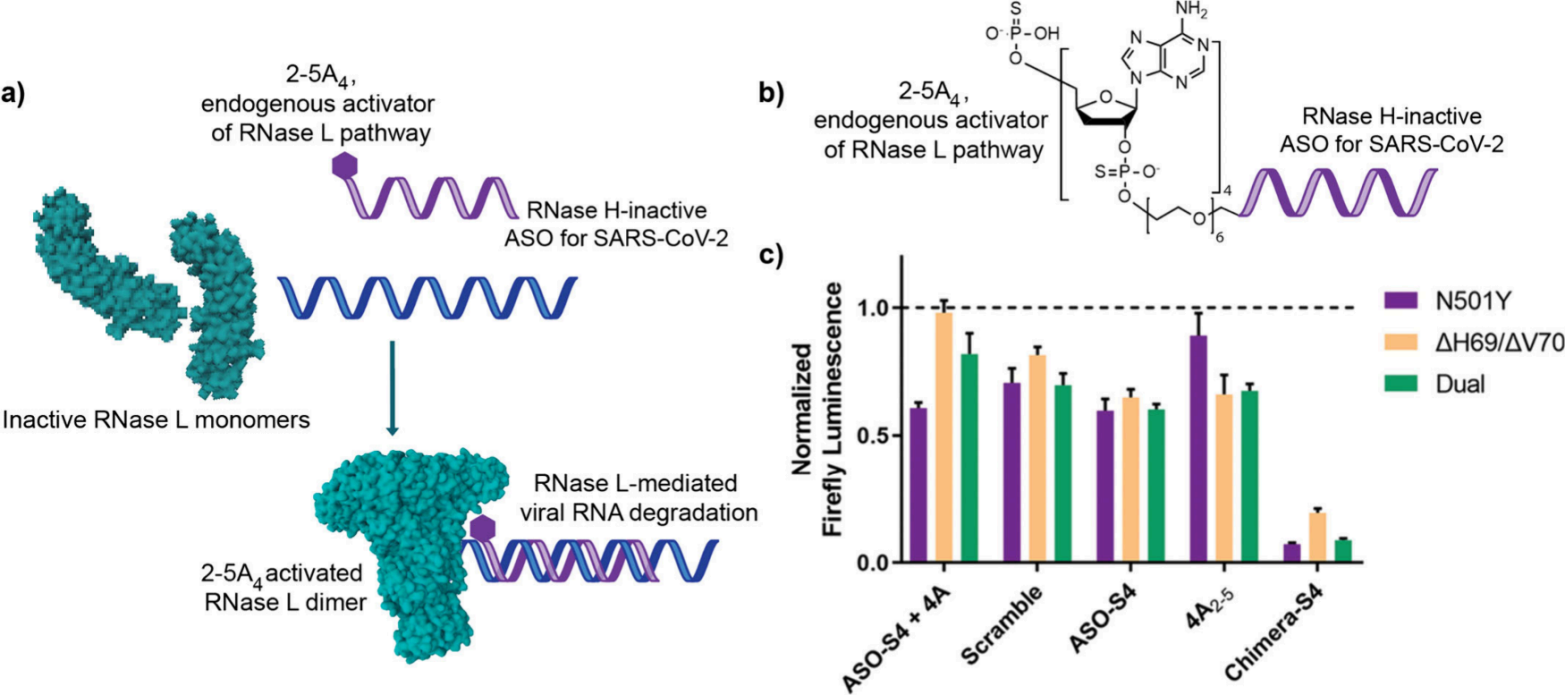
RNase L-activated inhibition of a pseudovirus infection model of SARS-CoV-2, using a 2′-5′ poly(A)_4_-modified ASO. a) Proposed mechanism of 2′-5′ poly(A)_4_ (2-5A_4_) mediated activation of RNase L for targeted viral RNA degradation. b) Chemistry of the 2-5A_4_–ASO conjugate for targeting SARS-CoV-2 RNA. c) Reduction in viral titer upon treatment with 2-5A_4_–ASO conjugate, measured using a luciferase infection model. Reprinted (adapted) with permission from ref (115). Copyright 2024 John Wiley and Sons, Inc.

**Figure 5:**
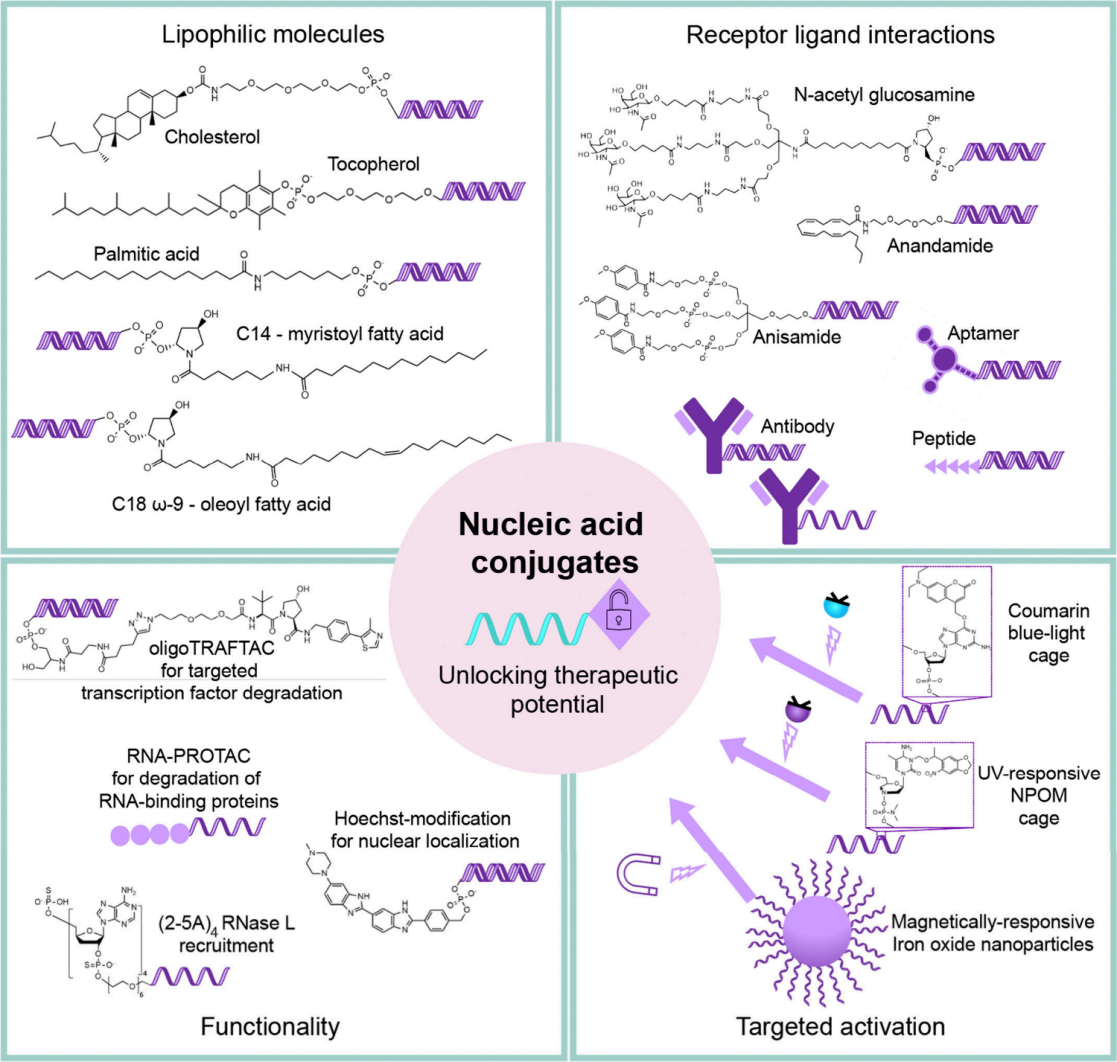
The development of nucleic acid conjugates. Next-generation therapeutic nucleic-acid conjugates can be broadly characterized into lipophilic conjugates, conjugates for receptor–ligand interactions, conjugates with new functionalities, and conjugates with targeted activation properties.

Chemical optimization of the modular components of nucleic acids has enabled nucleic acid therapeutics to breakthrough into the clinic. However, the current generation of these modifications has not seen the broadening of their applicability, with nucleic acid therapeutics stuck on liver and muscle diseases.^2^ The latest generation of nucleic acid therapeutics has contained a conjugate, a covalent attachment of another molecule, to improve cellular uptake. The literature is now awash with nucleic acid conjugates with an array of molecules from small molecules to large biomolecules. In this review, we will discuss the development of conjugates designed to improve the delivery, specificity, and targeting of nucleic acids and enhance or alter their functionality (Figures 2**-**5). These conjugates have the potential to enable nucleic acid therapeutics to treat more diseases in different areas of the body.

### Chemical Strategies for the Preparation of Oligonucleotide Conjugates

Two main chemical strategies^57,58^ are used for convalent linking of oligonucleotide with other molecules: presynthetic and postsynthetic strategies.

In the presynthetic approach, phosphoramidite monomers (the building blocks used in oligonucleotide solid phase synthesis) are modified to contain the desired molecule conjugate and incorporated into the growing oligonucleotide chain on a solid support. These modified nucleotides are incorporated directly during the standard phosphoramidite-based oligonucleotide synthesis before deprotection, release, and purification. This method allows for flexibility with attachment points, modifications at the 3′ or 5′ ends, or even a change between consecutive nucleotides. The most convenient strategy is to attach small molecule groups as presynthesized phosphoramidites at the 5′ end. Modifying the 3′ end is more challenging because the growing oligonucleotide is anchored to the solid support at that 3′ end; the bulky support matrix creates steric hindrance and limits reagent diffusion, reducing modification efficiency. The downsides of this approach lie in the challenging synthesis of the modified phosphoramidites.

In the postsynthetic approach, a small reactive functional group handle (such as an amine or thiol) is introduced into the oligonucleotide during solid-phase synthesis. These handles can be positioned at the 3′ or 5′ ends or on the actual nucleobases. This purified oligonucleotide (with a reactive handle) can then be coupled with the desired small molecule in solution. Since postsynthetic modifications are introduced after oligonucleotide synthesis, a broader range of functional groups and conditions can be employed. This approach, due to its modular nature, allows for separate synthesis and optimizations of the oligonucleotide and desired small molecule reagent. Furthermore, reactive handles incorporated into oligonucleotides are inexpensive and commercially available. A major disadvantage, however, is the requirement of handling the water-soluble oligonucleotide with another, often lipophilic, molecule, in solution, with potential solubility and stability issues. There are also a limited number of compatible reactions for coupling with varying yields and incomplete conversions due to competing side products; click chemistry reactions are commonly employed to get around this.

Examples discussed in this review rely upon both presynthetic^57,62,64,105^ and postsynthetic^59,75,83,111^ conjugation strategies.

### Conjugates for Delivery

Despite decades of chemical optimization, nucleic acid delivery continues to be a significant challenge. In comparison to small molecule drugs that can simply diffuse across lipophilic membranes based on concentration gradients, large polyanionic nucleic acids rely upon endocytosis for entry, trapping about 99% nucleic acids into endosomes.^59^ Productive uptake of nucleic acids requires the escape from the late endosome prior to lysosomal fusion and complete degradation. Thus, endosomal escape remains a key bottleneck in nucleic acid delivery.^60^ In addition to target engagement and endosomal trapping, accumulation in the lung, liver, and spleen remains a significant problem for nucleic acid delivery for both toxicity and tissue delivery. A detailed discussion on clinical dosing and related considerations for achieving effective therapeutic concentrations in desired tissues can be found elsewhere.^61^ A multitude of conjugates of nucleic acids, i.e., nucleic acids covalently linked with small and large molecules, have been explored to address these challenges (Figures 2**-**5).

#### Small Molecules for Receptor-Mediated Uptake

The state-of-the-art nucleic acid drugs today use targeting moieties or ligands, such as N-Acetylgalactosamine (GalNAc) for increased uptake to liver cells (Figure 1d). Clinically approved siRNA drugs lumasiran and givosiran that feature second-generation, 2′-OMe and 2′-F modifications are also covalent GalNAc conjugates. Eplontersen, a GalNAc-conjugated ASO (for treating Transthyretin amyloidosis), has also received FDA approval in 2024. GalNAc binds the asialoglycoprotein receptor or ASGPR – a highly expressed hepatic receptor with high recycling rates. This results in rapid endocytosis and robust RNAi responses in hepatocytes and associated pathologies. Currently only GalNAc conjugates have made it through clinical trials; however, alternative cases of small molecule receptor-mediated uptake have been demonstrated including anisamide, anandamide, and folate receptor-based targeting.

Anisamide is a ligand for sigma receptors, transmembrane proteins with roles in ion channel regulation, found on the endoplasmic reticulum and plasma membranes.^62^ Sigma receptors play key roles in cancer cell proliferation and tumor progression making them attractive targets. Moreover, they have high expression levels in various cancers and thus can be exploited for selective targeting. A triantennary anisamide moiety was converted into its phophoramidate form and incorporated directly via a DNA synthesizer. Trivalent-anisamide splice-switching ASO conjugates synthesized on the (full 2′-OMe backbone) showed a 2-fold increase in uptake and a 4-fold increase in target protein production over unmodified conjugates.^63^

Anandamide is a fatty acid neurotransmitter and an endogenous ligand for cannabinoid receptor 1 (CB1). CB1 has been implicated in a wide variety of neurological and cardiometabolic disorders, obesity, and cancers.^64^ Anandamide conjugates of various chemical architectures have been synthesized with varying potencies–all through postsynthetic conjugation. Simple monovalent conjugation^65^ results in a 2-fold increase in uptake while dendrimer siRNA-like structures with a single anandamide lipid and glucose enable knockdown in neural cells, while the unconjugated siRNA has no effect.^66^

Folate receptors are key glycoproteins overexpressed in cancer cells and play key roles in cancer cell metabolism.^67^ A folate-poly ethylene-glycol siRNA conjugate enabled gene silencing in an efficient and selective manner, exploring differences in folate receptor expression. The monovalent folate siRNA conjugate was able to knock down the target gene by over 75% while the unconjugated siRNA showed minimal activity.^68^

#### Lipophilic Molecules

The cell membrane is a negatively charged lipophilic barrier developed over millennia to prevent the entry of nucleic acids, which are large and negatively charged polymers. To hijack the membrane itself, conjugates of various lipid moieties have been employed to improve the overall lipophilicity and uptake profiles of the nucleic acids.

Cholesterol is a key component of lipid membranes, and vital in lipid metabolism and hormone synthesis.^69^ Cholesterol siRNA conjugates can be detected in heart, kidney, adipose, and lung tissue samples and, thus, have improved in vivo pharmacological properties. Cholesterol-siRNA conjugates induce RNAi-mediated target knockdown of greater than 50% while unconjugated siRNAs show little to no activity.^70^ Moreover, cholesterol ASO conjugates showed a 5-fold increase in potency in the muscle of rodents relative to unconjugated ASOs.^71^ In contrast, other lipophilic molecules such as palmitate and tocopherol-conjugated ASOs showed a significant increase in potency in the skeletal muscle of rodents and little enhancement in potency in monkeys.^71^ This study reveals the importance of chemistry and species-specific plasma protein binding profiles and their roles in efficacious delivery. In a different study, covalent cholesterol siRNA conjugates were preannealed with key lipoproteins such as HDL (high-density lipoprotein), LDL (low-density lipoprotein), and albumin. HDL preannealed complexes resulted in 5–18-times greater target gene reduction.^72^ They also showed how lipid chain length plays a key role in delivery, shorter chain fatty-acid conjugates, such as lauroyl (C12), myristoyl (C14) and palmitoyl (C16) did not reduce target gene levels in mouse livers; however, longer, alkyl chain, fatty acid conjugates such stearoyl (C18) and docosanyl (C22), significantly lowered target mRNA levels. In an alternate study, conjugates with a dendritic long-chain alkyl phosphate ester were preannealed to Human Serum Albumin (HSA) for improving their pharmacological profiles by increasing nuclease resistance and reducing nonspecific uptake by immune cells (macrophage pinocytosis).^73^

The efficacy and uptake of GalNAc and lipophilic targeting moieties such as tocopherol and cholesterol have been compared in hepatocytes. GalNAc was shown to increase uptake by 7-fold and efficacy by 10-fold, while cholesterol and tocopherol increased uptake 3–5-fold and efficacy 5-fold, all compared to the unconjugated ASO.^74^

Beyond hepatic delivery as in the above case, cholesterol and tocopherol-ASO heteroduplex conjugates (Figure 2a) have achieved CNS (central nervous system) delivery, particularly in neurons and microglial cells, inducing over a 5-fold knockdown of MALAT1 compared to free ASO duplex (Figure 2b).^75^ Another study used a 2′-O-hexadecyl (C16) chain (rather than the above terminal modifications) for effective and safe silencing in rodent CNS, eyes, and lungs.^76^ The study showed that their platform, the 2′-O-C16 siRNA in an Alzheimer’s mouse model, caused effective knockdown of amyloid-beta precursor protein (APP) in the CNS to alleviate physiological defects and normalize behavioral deficits.

#### Peptides and Antibodies

Small-molecule ligands can facilitate delivery of nucleic acids to many cell types, even those once considered impervious to uptake. Alternatively, peptides and, in particular, antibodies that act as ligands by binding to specific receptors and antigens, respectively, have exceptional targeting capabilities allowing for extrahepatic delivery.

#### Antibody–Oligonucleotide Conjugates

Nucleic acid conjugates with a variety of antibody fragments have been employed for extrahepatic delivery for CNS disorders, cardiac and muscle cancers, breast cancers, lung cancers, and leukemias.^77^

Transferrin receptors (TfR) regulate iron metabolism within cells. Their high physiological expression on brain endothelial cells across the blood–brain barrier (BBB), makes them exciting targets for CNS delivery. Antibody–ASO covalent conjugates directed toward murine TfR showed high bioavailability in the mouse brain reaching therapeutically relevant concentrations and extending survival of severely affected mouse SMA (spinal muscular atrophy) models.^78^ Moreover, enhanced uptake was also detected in other peripheral tissues such as muscle, which is particularly relevant in the case of neuromuscular diseases, such as SMA. Another study designed an oligonucleotide transport vehicle, an engineered Fc fragment with low-affinity binding for human TfR, for enhanced delivery across the BBB in mouse and nonhuman primates.^79^

Glioblastomas are highly aggressive brain cancers^80^ that contain cell surface markers/receptors such as CD44 (neural stem cell marker) and EphA2 (a key component of cell–cell signaling, overexpressed in cancers). These receptors have been exploited for targeted antibody-based delivery. Antibody–ASO conjugates directed toward CD44 and EphA2 allowed for effective internalization and targeting, and knockdown of the target.^81^ An ASO that targets the transcription factor MYC-associated factor X dimerization protein 3 (MXD3, key to leukemia survival) has also been developed via conjugation to an anti-CD22 antibody for targeting precursor B-cell acute lymphoblastic leukemias.^82^ Precursor B-cell (preB) acute lymphoblastic leukemia (ALL) is the most common type of ALL, with poor prognosis and limited treatments for both adults and children.^83^ This study showed improved survival rates for preB-ALL in xenografts and mouse models through targeted knockdown via the CD22 surface marker.

#### Peptide–Oligonucleotide Conjugates

Cell-penetrating peptides (CPPs) can enable improvements in cellular uptake, tissue targeting, and efficacy of nucleic acids therapeutics. There has been substantial work in developing CPP-conjugates with nucleic acids.^84^

First-generation CPPs such as Tat and its derivatives are polycationic in nature comprising mostly arginine and lysine residues, which allow for effective internalization. A TAT-siRNA conjugate was shown to undergo efficient RISC activity and perinuclear localization.^85^ While the cellular infiltration ability of the cationic peptides correlates with the numbers of arginine residues,^86^ the increase in the amount of such positive residues has been shown to result in cytotoxicity and renal toxicity in mouse models,^87^ presumably due to membrane disruption. Moreover, toxicity can arise from these arginine-rich CPPs coating free intracellular nucleic acids, leading to a generalized displacement of DNA- and RNA-binding proteins from chromatin and mRNA.^88^ Thus, efforts to generate CPPs with a more balanced charge led to the development of peptides, such as Pip5. The Pip5 series of CPPs comprises two arginine-rich regions with a varied central hydrophobic core. This series of CPPs, particularly, Pip5e and other subsequent series like Pip6, have been conjugated to PMOs for DMD, and have restored dystrophin protein in all muscle types, including cardiac tissues, with a single dose in mice DMD models.^89^

A peptide ligand has been used to target GLP-1 receptors in pancreatic β-cells. The GLP1-receptor is a crucial G-coupled protein receptor present in the brain and pancreatic cells and plays an important role in glucose metabolism. Capitalizing on GLP1-receptor expression, a peptide–ASO conjugate showed significant activity in mouse pancreatic β-cells, cells assumed to be resistant to ASO uptake.^90^

#### Nucleic Acid Aptamers for Delivery

Aptamers are a class of targeting molecules that can be engineered to target a wide variety of proteins, with affinities similar to those of antibodies.^15^ In one study, a single phosphorothioate chemical modification within the backbone of an aptamer resulted in a 1000-fold increase in affinity of the RNA aptamers against VEGF and thrombin, with binding affinities reaching 1–2 pM.^91^ In comparison, antibodies usually have an affinity in the range of 10–100 pM.^92^

The nucleolin aptamer, AS1411, has been shown to internalize into multiple cancer cell types, prostate, lung, and pancreas, and migrate to localize in the nucleus, targeting nucleolin, an important nucleolar phosphoprotein. An AS1411-splice switching oligonucleotide (SSO) conjugate was twice as effective than the free SSOs.^93^ The A10 aptamer was designed to target the prostate-specific membrane antigen (PSMA) and conjugates with siRNAs have been used for tissue-type specific delivery to prostate cancer cells.^94^ The A10-siRNA conjugate mediated tumor regression in a prostate cancer xenograft model. PSMA, the aptamer target, is also expressed in the solid tumor vasculature, making it a particularly promising target.

Aptamers are promising targeting molecules; as nucleic acids themselves, they are cost-effective and easy to synthesize and conjugate to nucleic acid therapeutics. However, they possess the same limiting factors for delivery and efficacy as nucleic acid therapeutics themselves.^17^

### Conjugates for Targeted Activation

The main focus of nucleic acid therapeutic targeting has traditionally been targeted delivery (as explained in the previous section), which involves biasing the delivery of a therapeutic agent to specific tissues. This approach aims to maximize the therapeutic effect by increasing the therapeutic concentration in the desired tissue. A promising alternative and/or complementary strategy is targeted activation, where the drug remains inactive during systemic circulation and is only activated at the target (like a prodrug) (Figure 5).^95^ Targeted activation can be achieved in two ways: using an internal stimulus that is specific to a disease or cell type or remote control using an external stimulus. Targeted activation will be able to minimize toxicity in nontarget tissues by deactivating the drug outside the target area. This strategy will enhance the therapeutic index and lead to better patient outcomes.^96^

There is a large focus on generating noncovalent nanoparticles for internal activation by pH and redox sensitive moieties,^97,98^ but very little work with covalent conjugates. Furthermore, the drawback of using many internal stimuli is that they are often located at multiple sites around the body. For instance, using the lower pH of a tumor environment^99^ therapeutically will be difficult for nucleic acid drugs that would also be activated by the lower pH of the endosome^100^ of any cell type they enter. Enzyme-based activation utilizes a more specific stimulus than varied nonspecific pH values or redox potentials in different cellular environments. One study designed enzyme-activatable caged ASOs by cyclizing its two terminals through a cathepsin B substrate peptide–which was efficiently uncaged by capthesin with effective tumor inhibition in a PC-3 tumor mice model.^101^ Remote control is an exciting alternative as it would allow precise spatiotemporal control. Current methods of remote control of nucleic acids have focused on conjugating photocages, molecules that cleave when illuminated. Light provides unmatched spatiotemporal precision. Light of various wavelengths has been used to cage oligonucleotides.^102,103^ Ultraviolet (UV) photocaged ASOs have shown light-activated knockdown of fluorescent proteins in cell-free systems, cell lines, zebrafish (Danio rerio), and *Xenopus* frog embryos.^104^ In one study, UV-caged morpholinos (Figure 3a) were used to regulate the key developmental *chordin* gene. Upon UV-exposure, the chordin morpholino ASOs were activated resulting in disruption of endogenous *chordin* levels and severe phenotype deformities (Figure 3b). UV photocaged 2′-F-modified siRNAs have also been developed for remote control in cell lines and zebrafish embryos.^105^ An advantage of light is the ability to use different colors for orthogonal activation. This has been utilized to enable orthogonal activation of two different ASOs with blue and UV light.^106^ The main limitation of using short wavelengths of light for activation is that they have very poor tissue penetration for use in the body.^107^ However, there are examples of near-infrared activated siRNA, which may be applicable for activation under the skin.^108^

### Conjugates for Functionality

Antisense oligonucleotides (ASOs) dominate the market for approved nucleic acid drugs. However, they generally operate through two limited mechanisms: splice modulation and RNase H activity.^7^ The substrate for splice-modulating antisense oligonucleotides (ASOs), pre-mRNA is located within the nucleus, locked away behind two lipophilic membranes. Enhancing the nuclear localization of these ASOs could potentially improve their splicing efficacy. On the other hand, RNase H-active oligonucleotides target mature mRNA primarily found in the cytoplasm, where the level of RNase H expression is relatively low. Additionally, studies suggest that less than 0.3% of siRNA^109^ and approximately 1% of ASOs^110^ successfully escape the endosome (GalNAc-nucleic acid conjugates). While nucleic acid therapeutics do achieve target engagement and biological effects, by using conjugates to further enhance target engagement and develop new functional capabilities, we may unlock even greater potency (Figure 5).

RNase H-dependent knockdown and splice-switching mechanisms are largely localized to the nucleus.^111^ Increasing ASO localization in the nucleus by conjugation with Hoechst dyes has resulted in improved knockdown of MALAT1, a long noncoding RNA cancer target present in the nucleus, compared to free LNA gapmer.^112^ Hoechst dyes bind to the minor groove of double-stranded DNA with very strong binding constants between 1 and 10 nM. Greater amounts of Hoechst-ASO conjugates were identified in the nucleus compared to unconjugated ASOs, presumably due to Hoechst dye-DNA interactions.

An alternate mechanism of action for RNase-inactive ASOs was established by recruiting the RNase L pathway. The interferon-inducible 2′-5′-Oligoadenylate Synthetase (OAS)/RNase L system is a potent antiviral pathway activated in response to double-stranded RNA.^113^ The key effector protein of this pathway is the highly potent cytoplasmic RNase L. The endogenous RNase L activator, 2′-5′-adenylate (2-5A_4_),^114^ has been conjugated to various antisense oligonucleotides, harnessing RNase L (Figure 4a) for targeted viral RNA degradation of the SARS-Co-V spike protein and the Respiratory Syncytial Virus transcription elongation factor (RSV ETF). In the case of the SARS-Co-V spike protein, they conjugated 2-5A_4_ to an ASO with an RNase H-inactive backbone, 2′-OMe (Figure 4b).^115^ This conjugate showed significant knockdown compared to the unconjugated ASO or cotreatment of 2-5A_4_. The SARS-CoV-2 viral titer also showed the same trend (Figure 4c). For the RSV TEF, the 2-5A_4_–ASO conjugate resulted in a 3-fold reduction in viral titer compared to the unconjugated ASO.^116^ In both of these cases, the 2-5A_4_ ligand allowed for the activation of a biochemical RNA degradation pathway, the RNase L pathway with the same cellular localization as the substrate for degradation, and viral mRNA, allowing for more effective knockdown by the conjugate compared to the free ASO.

## Future Perspectives

There is an extensive list of biologically active small molecules. The continued exploration of their conjugation with nucleic acids will likely yield the next-generation of therapeutics.

There is constant development of targeted delivery approaches for different diseases. It will be exciting to incorporate this research with stimuli control, developing joint targeted delivery, and activation. This will bias delivery of the nucleic acid therapeutic to the target site while inactivating off-target binding and toxicity. However, to achieve useful targeted activation, we require the use of better stimuli. For instance, internal stimuli that are more disease specific, like hypoxia for tumors.^117^ Remote stimuli with better tissue penetration, such as ultrasound and magnetism, would be ideal. A new preprint has used silica-encapsulated iron oxide nanoparticles, and their property of magnetic hyperthermia in the presence of alternating magnetic fields to selectively release oligonucleotides, opening up exciting possibilities for remote activation within the body.^118^

Despite all of the challenges we have mentioned, nucleic acid therapeutics have been demonstrated to effectively target their intended sites, have minimal toxicity, and lead to improved patient outcomes, demonstrating their therapeutic benefits. However, by enhancing target engagement and integrating innovative functional chemical groups, we achieve even greater and broader efficacy. The field of chemical biology and drug development is entering a new era of heterobifunctionals. After decades of optimization, as of the end of 2022, over 20 PROTAC drugs have entered clinical trials.^119^ Nucleic acids exhibit picomolar binding affinities to their target RNAs, and when combined with powerful small molecule ligands such as PROTAC warheads, they have the potential to form highly effective heterobifunctionals. Work in this direction has included the development of oligonucleotide transcription factor targeting chimeras (oligoTRAFTACs), a TF-binding oligonucleotide conjugated to an E3 ligase-recruiting ligand.^120^ Two oncogenic transcription factors (TFs), c-Myc and brachyury have been degraded by proteosomal recruitment using an oligoTRAFTAC. Additionally, small RNA sequences that bind RNA-binding proteins, LIN28 and RBFOX1, have been made into RNA-PROTACs^121^ upon conjugation with an E3-recruiting peptide, resulting in proteosomal degradation of the target proteins. Ubiquitination and subsequent proteasomal degradation are highly dependent on the spatial proximity of the E3 ligase and target proteins. Thus, linker length is a critical determinant of the PROTAC efficacy and the nature of ternary complex formation. Proof-of-concept RNA-PROTACs do not show quantitative degradation, likely due to the absence of structure–activity relationship (SAR) studies aimed at optimizing linker length.

Why limit existing technologies to PROTACs? A wide range of small molecule ligands have been developed to target a variety of biologically significant proteins, from receptors to crucial effectors in RNA degradation pathways. Integrating these functionalities with the field of nucleic acid therapeutics could result in a synergistic effect, resulting in truly translational and transformative medicine.
